# Transcriptomic Diversity of Pediatric Acute Myeloid Leukemia Genetic Drivers Correlates With Clinical Outcome and Expression of Stemness‐Related Genes

**DOI:** 10.1002/cam4.71325

**Published:** 2025-11-03

**Authors:** Quenton Rashawn Bubb, Elena Sotillo, Rebecca M. Richards, Crystal L. Mackall, Tanja A. Gruber, Agnieszka Czechowicz

**Affiliations:** ^1^ Stem Cell Biology and Regenerative Medicine Graduate Program, Medical Scientist Training Program Stanford University School of Medicine Stanford California USA; ^2^ Department of Pediatrics, Division of Hematology, Oncology, Stem Cell Transplantation and Regenerative Medicine, Stanford University School of Medicine Stanford California USA; ^3^ Institute for Stem Cell Biology and Regenerative Medicine Stanford University School of Medicine Stanford California USA; ^4^ Stanford Cancer Institute, Stanford University School of Medicine Stanford California USA; ^5^ Department of Pediatrics University of Wisconsin‐Madison Madison Wisconsin USA; ^6^ Division of Blood and Stem Cell Transplantation and Cell Therapy, Department of Medicine Stanford University School of Medicine Stanford California USA

**Keywords:** pediatric AML, transcriptomic diversity, transcriptomics

## Abstract

**Background:**

Pediatric acute myeloid leukemia (pAML) is comprised of a diverse set of oncogenic drivers (ODs) that have been risk‐stratified to inform prognosis and therapeutic decision‐making. Despite proteomic, transcriptomic, genetic, and epigenetic characterization of the pAML landscape, questions still remain about why certain ODs have poorer prognoses than others.

**Methods:**

We analyze a large pAML bulk‐RNA dataset (*n* = 435) and organize ODs along an axis of transcriptomic diversity by calculating the Simpson Diversity Index (SDI) of individual ODs.

**Results:**

When comparing patients with low diversity ODs to patients with high diversity ODs, we observe poorer overall survival (HR = 1.877, 95% CI: 1.377–2.558, *p* = 0.0002) among patients harboring high diversity ODs in addition to an enrichment of stemness‐related genes. We observe poorer survival of patients with high diversity ODs even when comparing patients with similar transcriptomic profiles (HR = 3.443, 95% CI: 1.817–6.525, *p* = 0.0028).

**Conclusion:**

We identify a link between transcriptomic diversity, expression of stemness‐related genes, and clinical outcome. Higher transcriptomic heterogeneity exhibited by high diversity ODs warrants further attention when identifying patients who can benefit from novel or high‐intensity therapy.

**Abbreviations**
AML: acute myeloid leukemia
HDOD: high diversity oncogenic driver
LDOD: low diversity oncogenic driver
OD: oncogenic driver
SDI: Simpson diversity index

## 1 Introduction

Pediatric Acute Myeloid Leukemia (pAML) is a malignant clonal expansion of the hematopoietic stem and progenitor compartment that is initiated by a diverse set of molecular lesions [1]. To organize the heterogeneous pAML landscape, several efforts have characterized risk stratification approaches to guide clinical decision‐making based on the prominent genetic lesion or oncogenic driver (OD) [2, 3, 4], and leukemic stem cell transcriptomic signatures have helped provide molecular bases for why certain oncogenic drivers have poorer prognoses than others [5, 6]. Scores of clinical data and analyses of outcomes have also led to the identification of favorable, intermediate, and high‐risk ODs that enable clinicians to plan therapeutic strategies and prognosticate [7]. Despite this, questions remain around the heterogeneity within and between different ODs in pAML, which poses unique challenges in the design and validation of targeted therapies that leverage correlations between leukemic genotype and phenotype. Specifically, an organizing framework of ODs that exhibit significant interpatient homogeneity or heterogeneity can be useful for preclinical and clinical study of novel treatment approaches. Here, we analyze a large (*n* = 435) pediatric acute leukemia transcriptomic dataset [2] and quantitatively characterize transcriptomic heterogeneity within different ODs. We aimed to evaluate whether transcriptomic heterogeneity independently influences prognosis and the extent to which prognosis is governed by specific ODs. We propose that transcriptomic diversity can cooperate with risk stratification algorithms and inform therapeutic decision‐making.

## 2 Methods

### 2.1 Bioinformatic Analysis

Normalized bulk RNA‐seq data and deidentified patient demographics were previously compiled [1, 8, 9, 10, 11] and published in Fornerod et al. [2]. We enumerated the number of transcriptional identities determined in Fornerod et al. [2] within each OD and subsequently quantified the Simpson Diversity Index (SDI) [12] for each OD in R. Survival analyses were carried out in Prism. Where more than 2 survival curves are on the same plot, the *p*‐value describes the statistical significance of the overall difference between the survival curves. Hazard ratios with corresponding 95% confidence intervals were calculated using a log‐rank test in Prism. Multivariate Cox hazard analyses were carried out in R using the package *survivalAnalysis*. Circos plots were created utilizing the R package circlize [13]. Patient summary tables were generated using the R package *gtsummary* [14].

## 3 Results

**FIGURE 1:**
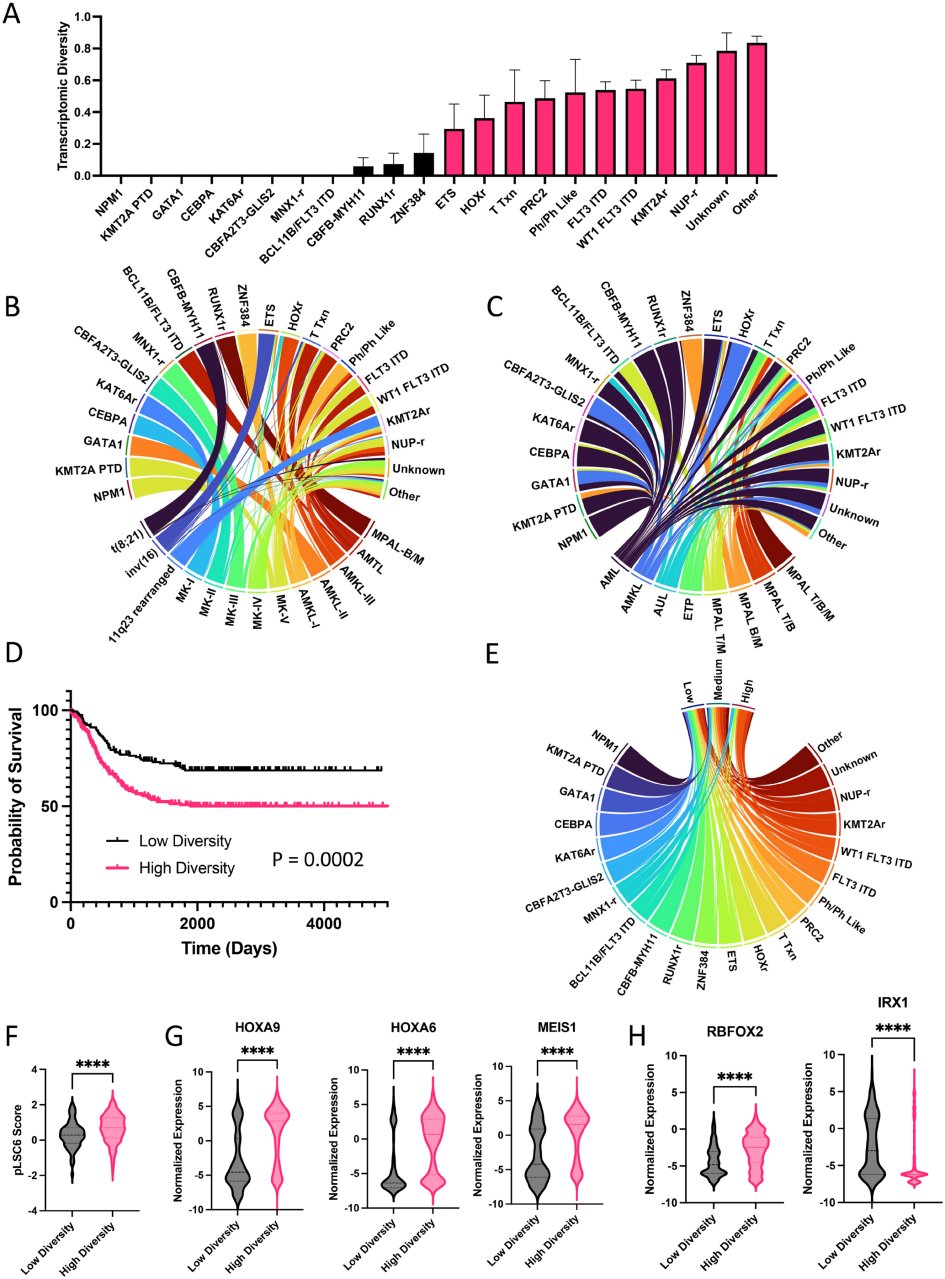
Analysis of Transcriptomic Diversity in pAML—(A) Transcriptomic Diversity (1—Simpson Diversity Index) of 22 Oncogenic Driver categories analyzed in Fornerod et al. [2]. Error bars indicate the standard deviation of each calculation of the Simpson Diversity Index. Low Diversity drivers are colored in black, while high diversity drivers are colored in magenta. (B) Circos plot highlighting the transcriptomic identity composition of each oncogenic driver. (C) Circos plot highlighting the leukemic subtype composition of each oncogenic driver. (D) Overall survival of patients with low diversity oncogenic drivers and high diversity oncogenic drivers (*p* = 0.0002, Log‐rank (Mantel‐Cox) test). (E) Circos plot highlighting the oncogenic driver composition of each pLSC6‐based risk category [2]. (F) Comparison of pLSC6 scores between low diversity and high diversity oncogenic drivers (****p = 0.0002, unpaired *t* test). (G) HOX‐family and MEIS1 expression among low diversity and high diversity oncogenic drivers (****p < 0.0001, unpaired *t* test). (H) RBFOX2 and IRX1 expression among low diversity and high diversity oncogenic drivers (****p < 0.0001, unpaired *t* test). AMKL, acute megakaryoblastic leukemia; AML, acute myeloid leukemia; AUL, acute undifferentiated leukemia; B/M, B‐lymphoid and myeloid co‐expression; ETP, early T‐precursor; ITD, internal tandem duplication; MK, mixed karyotype; MPAL, mixed‐phenotype acute leukemia; Ph‐like, Philadelphia chromosome–like acute lymphoblastic leukemia; PTD, partial tandem duplication; T/B, T‐lymphoid and myeloid co‐expression; T/B/M, T‐lymphoid, B‐lymphoid, and myeloid co‐expression; T/M T‐lymphoid and myeloid co‐expression; Txn, transcription.

Among the 22 categories of ODs analyzed in the pediatric cohort, 13 transcriptomic “identities” or clusters were previously defined based on shared gene expression by Fornerod et al. (Table S1). We quantitatively assessed the transcriptomic heterogeneity by calculating the Simpson Diversity Index [12] (SDI) of individual ODs. In this context, the SDI enumerates the probability that two patients harboring the same OD will have the same transcriptomic identity. Thus, subtracting the SDI from 1 (1–SDI) yields the transcriptomic diversity of a given OD, where a value of 0 is interpreted as complete interpatient transcriptomic homogeneity, and a value of 1 is significant interpatient transcriptomic heterogeneity. We empirically set the threshold between low diversity ODs (LDODs) and high diversity ODs (HDODs) at the median transcriptomic diversity (0.219); thus, HDODs are those in which there is a less than 78.1% chance that two patients have the same transcriptional identity. With this analysis, ODs like NPM1 and GATA1 are transcriptomically homogeneous in this cohort, while other ODs, like NUP rearrangements (NUP‐r), are more heterogeneous (Figure 1A). This heterogeneity can be appreciated when visualizing the transcriptomic composition of each OD (Figure 1B). LDODs are comprised of single transcriptomic identities, while HDODs are comprised of several different transcriptomic identities. Strikingly, the leukemic subtype composition of each OD does not reflect underlying transcriptomic heterogeneity (Figure 1C). Notably, pAML is the dominant leukemic subtype for LDODs like NPM1 and HDODs like NUP‐r, despite significant underlying transcriptomic heterogeneity in the latter. When comparing the overall survival (OS) of patients with LDODs in comparison to patients with HDODs, we observe that patients harboring HDODs have poorer prognoses (HR = 1.877, 95% CI: 1.377–2.558, *p* = 0.0002, Figure 1D). When observing the spectrum of LDODs and HDODs within risk categories defined by pLSC6 [6] scores, we observe that HDODs occupy a large proportion of medium and high‐risk categories [2] (Figure 1E) and have higher pLSC6 scores (Figure 1F). We found that expression of HOX‐family genes and leukemic collaborating gene MEIS1 [15] are enriched in HDODs (Figure 1G). Additionally, RBFOX2, a gene found to be critical in leukemic stem cell self‐renewal and chromatin modifier [16], is upregulated in HDODs, while IRX1, a negative regulator of HOX expression [17], is enriched in LDODs (Figure 1H). Multivariate analyses showed that when accounting for leukemic subtype and pLSC6 score, HDODs were associated with poor overall survival (HR = 1.49, 95% CI: 1.05–2.11, *p* = 0.025, Figure S1). In an analysis of deviance, the inclusion of both OD identity and the derived HDOD/LDOD classification did not improve model fit (log‐likelihood = −869.46; *χ* ^2^ = 0, df = 0, *p* = 1.0, Table S2), highlighting collinearity between our transcriptomic diversity axis and the OD categories. For further comparison of prognostic value between the binary HDOD/LDOD classifier and OD categories, we assessed model fit using the Akaike Information Criterion (AIC). The OD‐based model provided a substantially better fit than the transcriptomic diversity‐based model (ΔAIC = 11.97, Table S3), indicating that specific oncogenic drivers capture more prognostic information.

**FIGURE 2:**
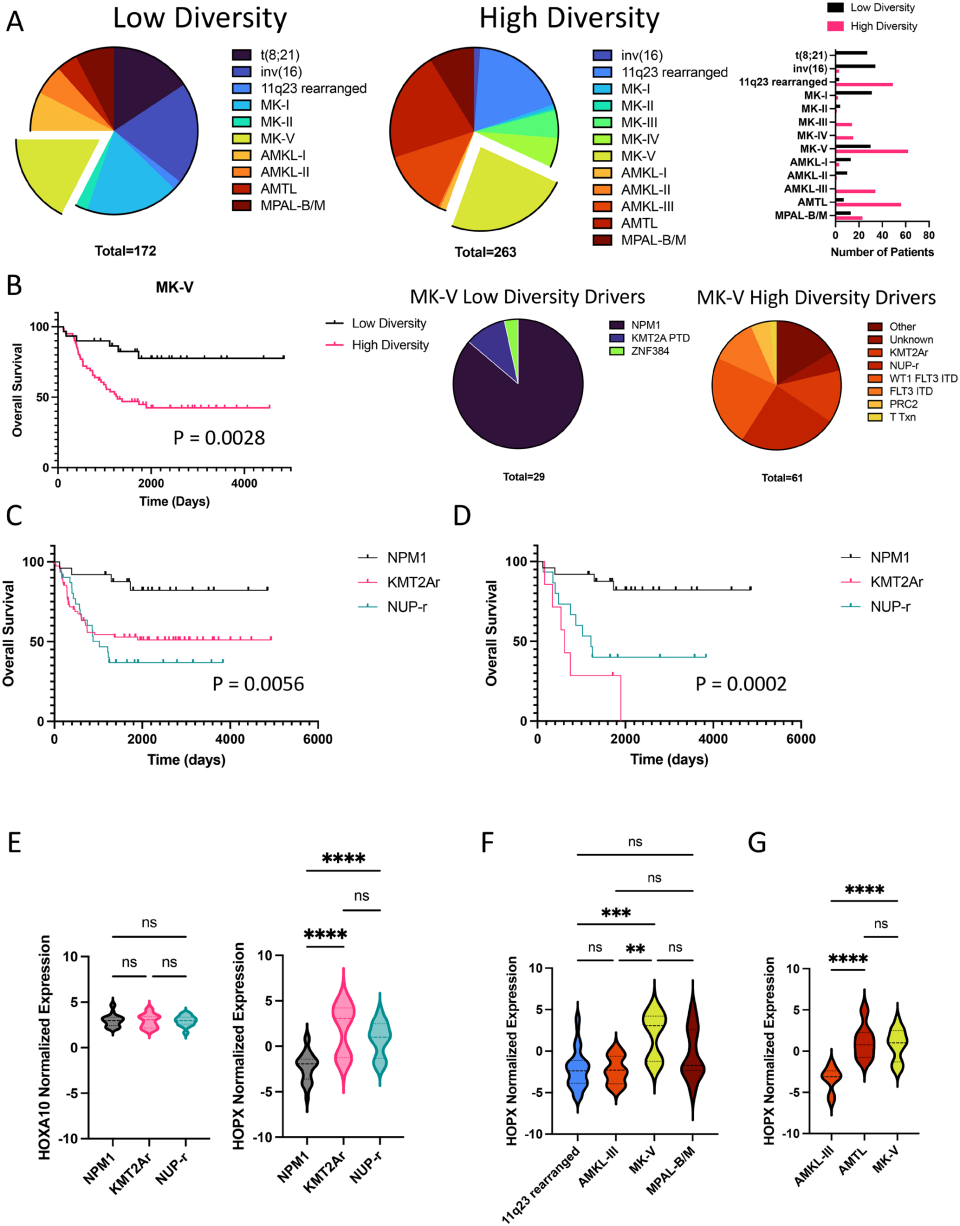
Diverse oncogenic drivers modify the probability of survival despite transcriptomic similarity. (A) Proportions of transcriptomic identities within low and high diversity oncogenic drivers (pie charts) and the distribution of patients across transcriptomic identities (bar plot). (B) Overall survival of patients with low diversity oncogenic drivers and high diversity oncogenic drivers (proportions shown in pie charts) (*p* = 0.0028, Log‐rank (Mantel‐Cox) test). (C) Overall survival of patients harboring NPM1, KMT2Ar, and NUP‐r in the entire pediatric cohort (*p* = 0.0056, Log‐rank (Mantel‐Cox) test). (D) Overall survival of patients harboring NPM1, KMT2Ar, and NUP‐r within the MK‐V transcriptomic cluster (*p* = 0.0002, Log‐rank (Mantel‐Cox) test). (E) HOX10 and HOPX expression among patients harboring NPM1, KMT2Ar, and NUP‐r within the MK‐V transcriptomic cluster (****p < 0.0001, One‐way ANOVA). (F) HOPX expression among patients harboring KMT2A rearrangements across transcriptomic identities (**p = 0.0037, ***p = 0.0002, One‐way ANOVA). (G) HOPX expression among patients harboring NUP rearrangements across transcriptomic identities (****p < 0.0001, One‐way ANOVA).

When visualizing the proportions of transcriptional identities within LDODs and HDODs, we note comparable numbers of patients within the MK‐V transcriptional identity (Figure 2A). When isolating the MK‐V transcriptional identity, patients with HDODs underlying MK‐V had significantly poorer OS (HR = 3.443, 95% CI: 1.817–6.525, *p* = 0.0028, Figure 2B). This was a surprising result, considering that LDODs and HDODs within this transcriptomic cluster have similar differentially expressed genes in this dataset. In the bulk dataset, considering specific ODs that are represented in the MK‐V cluster, we found that patients with HDODs KMT2Ar (HR = 3.787, 95% CI: 1.912–7.501, *p* = 0.0064) and NUP‐r (HR = 5.474, 95% CI: 2.412–12.42, *p* = 0.0004) had worse OS in comparison to patients with the dominant LDOD, NPM1 (*p* = 0.0056, Figure 2C). This was also true when only considering patients that harbor the NPM1, KMT2Ar (HR = 8.694, 95% CI: 1.511–50.01, *p* < 0.0001), and NUP‐r (HR = 5.045, 95% CI: 1.555–16.37, *p* = 0.0025) ODs within the MK‐V category, implying a deeper role for the ODs themselves beyond their effects on the transcriptome (*p* = 0.0002, Figure 2D). HDODs also occupy a larger proportion of mixed phenotype acute leukemias (MPALs) (Figure S2A), express higher levels of HOXA10 (Figure S2B), and patients harboring HDODs have poorer OS (HR = 2.495, 95% CI: 1.147–5.426, *p* = 0.0394) in comparison to patients with LDODs (Figure S2C). While the expression of the stem‐related gene HOXA10 is not significantly different between NPM1, KMT2Ar, and NUP‐r harboring leukemias, we found that HOPX expression is significantly different between KMT2Ar and NUP‐r in comparison to NPM1 (Figure 2E). HOPX is a stem cell marker that has been associated with poor prognosis in AML, but the functional role of HOPX has not yet been determined in the context of hematopoiesis [18]. We observed an enrichment of HOPX expression among KMT2Ar leukemias in the MK‐V transcriptional identity (Figure 2F) and among NUP‐r leukemias in the AMTL and MK‐V transcriptional identities (Figure 2G).

## 4 Discussion

In summary, we quantitatively assessed transcriptomic heterogeneity in a cohort of pediatric leukemia and discovered a spectrum of transcriptomic diversity across the different ODs. We highlight pAML heterogeneity at the transcriptomic level. Transcriptomic diversity correlated with poor prognosis (HR = 1.877, 95% CI: 1.377–2.558, *p* = 0.0002) and expression of stemness‐related genes. The observation that transcriptomic diversity is associated with poorer survival when controlling for pLSC6 and leukemic subtype (HR = 1.49, 95% CI: 1.05–2.11, *p* = 0.025) implies that transcriptomic diversity captures significant prognostic information that is not accounted for by the pLSC6 score alone. While OD identities provide more prognostic value, the HDOD/LDOD classifier provides a biologically interpretable summary axis that reduces the complexity of the diverse pAML landscape while retaining prognostic signal. In future studies, we hope to integrate other features that influence prognosis, such as treatment exposure, minimal residual disease, and single‐cell transcriptomic profiling to further delineate the limitations and influence of transcriptomic heterogeneity on prognosis. Our observations provide additional context to the underlying features of adverse‐risk leukemias, namely transcriptomic heterogeneity, and highlight the need for transcriptomic profiling to aid in the development, characterization, and validation of novel therapeutics and therapeutic strategies.

Our data synergizes with the pLSC6 score, a current risk‐determination tool, by adding a dimension that considers interpatient heterogeneity (Figure S3). This additional dimension further contextualizes ODs such as CBFA2T3::GLIS2, which have extremely poor overall survival [2] and medium‐high risk pLSC6 scores despite exhibiting low interpatient heterogeneity (Figure S3). Our data imply that interpatient transcriptional heterogeneity may not be predictable by genetic characterization of oncogenic drivers alone, and performing RNAseq at diagnosis would provide an oncogenic driver, a pLSC6 score, and transcriptional identity, which can yield more specific prognostic information. Future studies will focus on multimodal data integration (transcriptomic signature determination [3, 4, 6] and genetics‐based risk categorization [7]) into a single model and subsequent validation with a prospective clinical trial utilizing Clinical Laboratory Improvement Amendments (CLIA) certified transcriptome data. Our observations also highlight the importance of diverse preclinical models to account for interpatient transcriptomic variance in therapeutic development.

While the transcriptomic heterogeneity underlying HDODs is likely related to the diversity of genetic fusions [19, 20] and complex karyotypes within these ODs, we highlight a link between transcriptomic heterogeneity, stemness‐related genes, and prognosis. We also show that poor prognosis in a transcriptomic category of AML (MK‐V), previously shown to be enriched for primitive cell states [2], is driven by HDODs (HR = 3.443, 95% CI: 1.817–6.525, *p* = 0.0028). HDODs also constitute the majority of MPALs and are enriched in HOXA10 expression, consistent with our broader observation that transcriptomic heterogeneity is associated with these drivers. It is well established that patients with KMT2Ar and NUP‐r fusions have poorer prognoses in comparison to patients harboring ODs like NPM1 [2, 4].

Despite similar transcriptomes, including high expression of stemness‐related genes like HOXA10, there is a stark difference in overall survival when comparing LDOD NPM1 to HDODs NUP‐r (HR = 5.045, 95% CI: 1.555–16.37, *p* = 0.0025) and KMT2Ar (HR = 8.694, 95% CI: 1.511–50.01, *p* < 0.0001). We hypothesize that this is due to the biological consequences of the ODs themselves. NPM1 has been shown to maintain the pre‐established, active chromatin state of leukemic cells by inhibiting histone deacetylases and cannot directly alter chromatin structure for transcription initiation [21]. KMT2Ar and NUP‐r in‐frame fusions, however, can both activate transcription, namely of HOXA genes, and directly modify chromatin structure [22]. The epigenetic consequences of KMT2A or NUP gene rearrangements can confer fusion harboring cells with greater plasticity and chemoresistance. When minimizing transcriptional differences through observation of a single transcriptional identity, we observe higher expression of HOPX in KMT2Ar and NUP‐r leukemia. While the function of HOPX in human hematopoiesis is unknown, it has been shown to interact with histone deacetylase in murine models and regulates differentiation in various cell types [23].

Our analysis of transcriptional heterogeneity underscores the importance of targeted therapies that exploit biological vulnerabilities, such as the use of menin inhibitors in KMT2Ar or NPM1‐mutated leukemias [24], or combinatorial immunotherapeutic approaches [25] that span the diverse pAML landscape.

## 5 Conclusion

Interpatient transcriptomic heterogeneity at the bulk RNA‐seq level is associated with poor survival and expression of stemness‐related genes. This association remains observable when comparing LDODs and HDODs with similar bulk transcriptomic profiles, both implying a deeper role for the influence of ODs on response to therapy and necessitating novel treatment strategies to improve outcomes. Diagnostic transcriptomic profiling can enrich our understanding of the diverse pAML landscape, while aiding in the identification of adverse‐risk patients that require high‐intensity therapy.

## Author Contributions

**Quenton Rashawn Bubb:** conceptualization, investigation, writing – original draft, methodology, validation, writing – review and editing, formal analysis, visualization. **Elena Sotillo:** supervision, resources, writing – review and editing. **Rebecca M. Richards:** writing – review and editing, supervision, resources. **Crystal L. Mackall:** funding acquisition, writing – review and editing, supervision, resources. **Tanja A. Gruber:** resources, supervision, data curation, writing – review and editing, funding acquisition. **Agnieszka Czechowicz:** funding acquisition, writing – review and editing, resources, supervision, project administration.

## Conflicts of Interest

Q.R.B., E.S., R.M.R., C.L.M., and A.C. are inventors on a recently filed patent application related to separate CAR T‐related work. Additionally, A.C. discloses financial interests in the following entities working on antibody‐based conditioning approaches: Beam Therapeutics, Editas Medicines, GV, Inograft Biotherapeutics, Kyowa Kirin, and Prime Medicines. In addition, she is an inventor on antibody‐based conditioning patents licensed to Jasper Therapeutics, Gilead Sciences, Inograft Biotherapeutics, and Magenta Therapeutics. C.L.M. and E.S. are coinventors on multiple patents related to CAR T. C.L.M. is a cofounder, consults, and holds equity in CARGO Therapeutics, Link Cell Therapies, and GBM NewCo. C.L.M. consults for Ensoma and Immatics and received research funding from Tune and Lyell Immunopharma. E.S. consults for and holds equity in Lyell Immunopharma, and consults for Lepton Pharmaceuticals and Galaria.

## Supporting information


**Figure S1:** Forest plot summarizing a Multivariate Cox Survival Analysis of the pediatric cohort, in which Hazard Ratios (HR) with corresponding 95% confidence intervals (CI), and *p*‐values were calculated for each leukemic subtype relative to AML, pLSC6 scores, and diversity characterization of oncogenic drivers (HDODs vs. LDODs).
**Figure S2:** (A) Proportions of oncogenic drivers among MPALs (left) and proportions of low and high diversity oncogenic drivers among MPALs (right). (B) HOXA10 expression among low diversity and high diversity oncogenic drivers in MPAL (***p < 0.0006, unpaired t test). (C) Overall survival patients with MPAL who harbor low or high diversity oncogenic drivers (*p* = 0.0394, Log‐rank (Mantel‐Cox) test).
**Figure S3:** Circular box and whisker plot highlighting the pLSC6 scores and their corresponding risk strata for patients harboring HDODs and LDODs. Patients with calculated pLSC6 scores in the Medium and High risk categories are highlighted in red.

**Table S1:** Deidentified patient and treatment characteristics of pediatric bulk RNA seq cohort. AMKL, acute megakaryoblastic leukemia; AML, acute myeloid leukemia; AUL, acute undifferentiated leukemia; B/M, B‐lymphoid and myeloid co‐expression; ETP, early T‐precursor; ITD, internal tandem duplication; MK, mixed karyotype; MPAL, mixed‐phenotype acute leukemia; Ph‐like, Philadelphia chromosome–like acute lymphoblastic leukemia; PTD, partial tandem duplication; T/B, T‐lymphoid and myeloid co‐expression; T/B/M, T‐lymphoid, B‐lymphoid, and myeloid co‐expression; T/M T‐lymphoid and myeloid co‐expression; Txn, transcription. Demographic information adapted from Fornerod et al. [2].

**Table S2:** Analysis of deviance comparing nested Cox proportional hazards models that incorporate OD, pLSC6 score, and Immunophenotype versus OD, pLSC6 score, Immunophenotype, and Transcriptomic Diversity (HDOD or LDOD). NA, Not Applicable.

**Table S3:** Model comparison using Akaike Information Criterion (AIC).

## Data Availability

Normalized bulk RNA‐seq data comprised previously published data and is available in its respective sources noted in Fornerod et al. [2]. All other data and analyses utilized in support of the findings of this study may be made available from the corresponding author upon reasonable request.
