# Supplementary material for: Transcriptomic Diversity of Pediatric Acute Myeloid Leukemia Genetic Drivers Correlates With Clinical Outcome and Expression of Stemness‐Related Genes
Source: Cancer Med. 2025 Nov 3;14(21):e71325. doi: 10.1002/cam4.71325 (PMC12580620; doi:10.1002/cam4.71325)
Supplement: Supplementary file 1 — Figure S1: Forest plot summarizing a Multivariate Cox Survival Analysis of the pediatric cohort, in which Hazard Ratios (HR) with corresponding 95% confidence intervals (CI), and p‐values were calculated for each leukemic subtype relative to AML, pLSC6 scores, and diversity characterization of oncogenic drivers (HDODs vs. LDODs). Figure S2: (A) Proportions of oncogenic drivers among MPALs (left) and proportions of low and high diversity oncogenic drivers among MPALs (right). (B) HOXA10 expression among low diversity and high diversity oncogenic drivers in MPAL (***p < 0.0006, unpaired t test). (C) Overall survival patients with MPAL who harbor low or high diversity oncogenic drivers (p = 0.0394, Log‐rank (Mantel‐Cox) test). Figure S3: Circular box and whisker plot highlighting the pLSC6 scores and their corresponding risk strata for patients harboring HDODs and LDODs. Patients with calculated pLSC6 scores in the Medium and High risk categories are highlighted in red. [file CAM4-14-e71325-s002.pdf]

Endpoint	Subgroup	n	HR	CI	p
Overall Survival	MPAL T/M vs AML (Ref)	409	0.54	(0.25–1.16)	0.113
	AUL vs AML (Ref)		0.88	(0.12–6.31)	0.896
	AMKL vs AML (Ref)		1.26	(0.81–1.97)	0.301
	MPAL B/M vs AML (Ref)		1.34	(0.76–2.34)	0.311
	HDOD vs LDOD (Ref)		1.49	(1.05–2.11)	0.025
	pLSC6 Score		1.74	(1.41–2.16)	<0.001
	MPAL T/B vs AML (Ref)		2.91	(0.71–11.92)	0.137
	MPAL T/B/M vs AML (Ref)		4.84	(1.51–15.50)	0.008

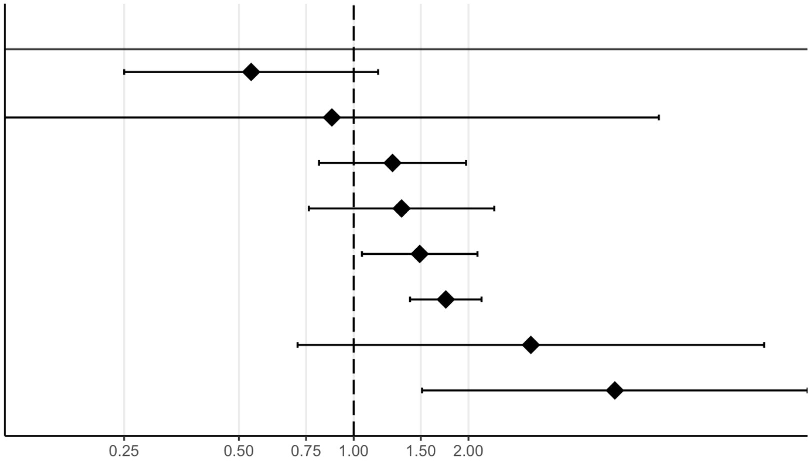

Supplementary Figure 1. Forest plot summarizing a Multivariate Cox Survival Analysis of the pediatric cohort, in which Hazard Ratios (HR) with corresponding 95% confidence intervals (CI), and p-values were calculated for each leukemic subtype relative to AML, pLSC6 scores, and diversity characterization of oncogenic drivers (HDODs vs. LDODs).

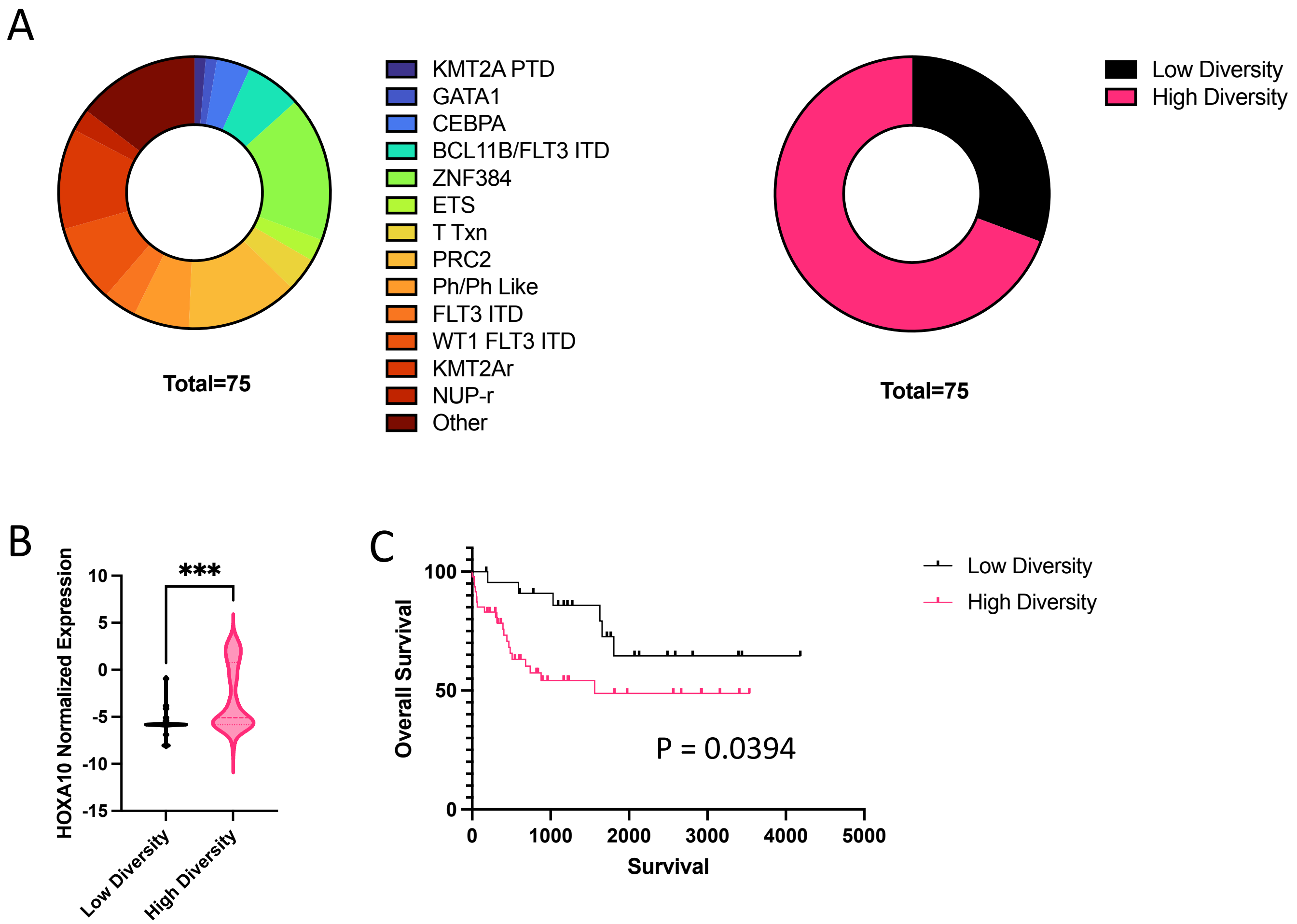

Supplementary Figure 2. (A) Proportions of oncogenic drivers among MPALs (left) and proportions of low and high diversity oncogenic drivers among MPALs (right). (B) HOXA10 expression among low diversity and high diversity oncogenic drivers in MPAL (***: P < 0.0006, unpaired t test). (C) Overall survival patients with MPAL who harbor low or high diversity oncogenic drivers (P = 0.0394, Log-rank (Mantel-Cox) test).

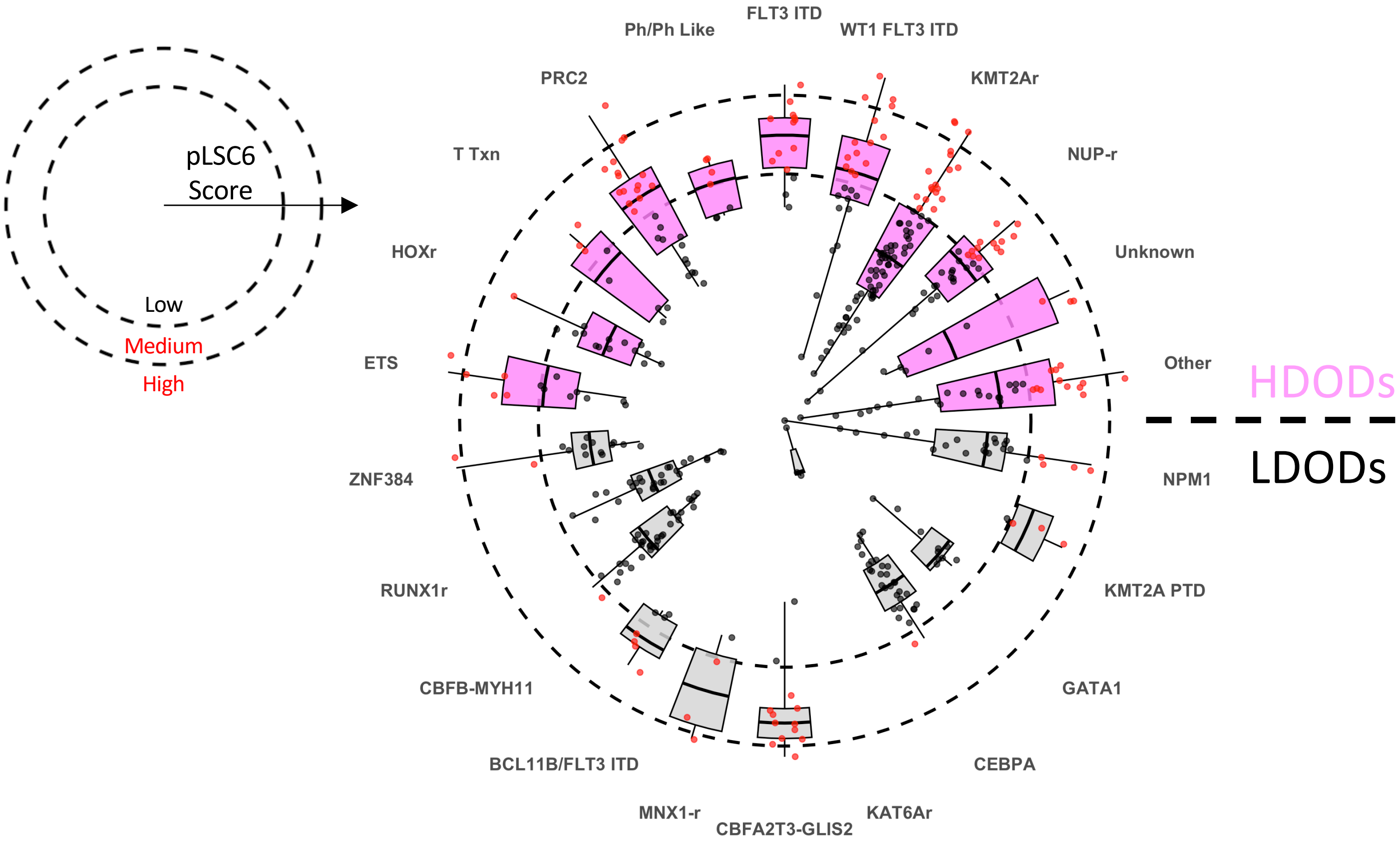

Supplementary Figure 3. Circular box and whisker plot highlighting the pLSC6 scores and their corresponding risk strata for patients harboring HDODs and LDODs. Patients with calculated pLSC6 scores in the Medium and High risk categories are highlighted in red.
