# Supplementary material for: Transcriptomic Diversity of Pediatric Acute Myeloid Leukemia Genetic Drivers Correlates With Clinical Outcome and Expression of Stemness‐Related Genes
Source: Cancer Med. 2025 Nov 3;14(21):e71325. doi: 10.1002/cam4.71325 (PMC12580620; doi:10.1002/cam4.71325)
Supplement: Supplementary file 2 — Table S1: Deidentified patient and treatment characteristics of pediatric bulk RNA seq cohort. AMKL, acute megakaryoblastic leukemia; AML, acute myeloid leukemia; AUL, acute undifferentiated leukemia; B/M, B‐lymphoid and myeloid co‐expression; ETP, early T‐precursor; ITD, internal tandem duplication; MK, mixed karyotype; MPAL, mixed‐phenotype acute leukemia; Ph‐like, Philadelphia chromosome–like acute lymphoblastic leukemia; PTD, partial tandem duplication; T/B, T‐lymphoid and myeloid co‐expression; T/B/M, T‐lymphoid, B‐lymphoid, and myeloid co‐expression; T/M T‐lymphoid and myeloid co‐expression; Txn, transcription. Demographic information adapted from Fornerod et al. [2]. [file CAM4-14-e71325-s003.docx]

| **Combined Cohort** | **N = 435***^1^* |
| --- | --- |
| **Treatment Protocol** |  |
| AAML03P1 | 52 (12%) |
| AAML0531 | 88 (20%) |
| AIEOP AML 2002 | 22 (5.1%) |
| AML02 | 46 (11%) |
| AML08 | 22 (5.1%) |
| ANLL92 | 1 (0.2%) |
| ANLL94 | 2 (0.5%) |
| ANLL97 | 1 (0.2%) |
| ANLL97 P3 | 1 (0.2%) |
| ANLL97 S14 | 1 (0.2%) |
| BFM04 | 14 (3.2%) |
| BFM12 | 1 (0.2%) |
| CCG-2961 | 19 (4.4%) |
| DB-AML01 SR | 1 (0.2%) |
| MRC15 | 3 (0.7%) |
| MRC15 SR | 1 (0.2%) |
| NOPHO AML 2004 | 13 (3.0%) |
| NOPHO AML12 | 1 (0.2%) |
| NOPHO-DBH AML | 2 (0.5%) |
| Not Reported | 144 (33%) |
| **Key Oncogenic Driver Event** |  |
| BCL11B/FLT3 ITD | 7 (1.6%) |
| CBFA2T3-GLIS2 | 13 (3.0%) |
| CBFB-MYH11 | 34 (7.8%) |
| CEBPA | 31 (7.1%) |
| ETS | 13 (3.0%) |
| FLT3 ITD | 14 (3.2%) |
| GATA1 | 10 (2.3%) |
| HOXr | 15 (3.4%) |
| KAT6Ar | 3 (0.7%) |
| KMT2A PTD | 4 (0.9%) |
| KMT2Ar | 78 (18%) |
| MNX1-r | 4 (0.9%) |
| NPM1 | 25 (5.7%) |
| NUP-r | 34 (7.8%) |
| Other | 33 (7.6%) |
| Ph/Ph Like | 7 (1.6%) |
| PRC2 | 28 (6.4%) |
| RUNX1r | 27 (6.2%) |
| T Txn | 8 (1.8%) |
| Unknown | 8 (1.8%) |
| WT1 FLT3 ITD | 25 (5.7%) |
| ZNF384 | 14 (3.2%) |
| **Immunophenotype** |  |
| AMKL | 53 (12%) |
| AML | 283 (65%) |
| AUL | 5 (1.1%) |
| ETP | 19 (4.4%) |
| MPAL B/M | 37 (8.5%) |
| MPAL T/B | 4 (0.9%) |
| MPAL T/B/M | 3 (0.7%) |
| MPAL T/M | 31 (7.1%) |
| **FAB Designation** |  |
| M0 | 11 (2.5%) |
| M1 | 52 (12%) |
| M1eo | 1 (0.2%) |
| M2 | 61 (14%) |
| M4 | 72 (17%) |
| M5 | 54 (12%) |
| M5a | 5 (1.1%) |
| M6 | 3 (0.7%) |
| M6a | 1 (0.2%) |
| M7 | 52 (12%) |
| Unknown | 123 (28%) |
| **pLSC6 Category** |  |
| Low | 302 (69%) |
| Medium | 119 (27%) |
| High | 14 (3.2%) |
| **Stem Cell Transplant in First Remission** |  |
| Yes | 86 (20%) |
| No | 246 (57%) |
| Unknown | 103 (24%) |
| **Reference Group** |  |
| Blood Cancer Discovery. 2021 Nov 1;2(6):586-99. | 132 (30%) |
| Nat Genet. 2017 Aug;49(8):1211-1218 | 19 (4.4%) |
| Nat Genet. 2017 Mar;49(3):451-456 | 45 (10%) |
| Nat Med. 2018 Jan;24(1):103-112 | 159 (37%) |
| Nature. 2018 Oct; 562(7727): 373-379. | 80 (18%) |
| *^1^* n (%) | |

**Supplementary Table 1.** Deidentified patient and treatment characteristics of pediatric bulk RNA seq cohort. PTD, partial tandem duplication; ITD, internal tandem duplication; Txn, transcription; Ph-like, Philadelphia chromosome–like acute lymphoblastic leukemia; MK, mixed karyotype; AMKL, acute megakaryoblastic leukemia; AML, acute myeloid leukemia; AUL, acute undifferentiated leukemia; ETP, early T-precursor; MPAL, mixed-phenotype acute leukemia; B/M, B-lymphoid and myeloid co-expression; T/B, T-lymphoid and myeloid co-expression; T/B/M, T-lymphoid, B-lymphoid, and myeloid co-expression; T/M T-lymphoid and myeloid co-expression. Demographic information adapted from Fornerod et. al^1.^
