# Supplementary material for: Transcriptomic Diversity of Pediatric Acute Myeloid Leukemia Genetic Drivers Correlates With Clinical Outcome and Expression of Stemness‐Related Genes
Source: Cancer Med. 2025 Nov 3;14(21):e71325. doi: 10.1002/cam4.71325 (PMC12580620; doi:10.1002/cam4.71325)
Supplement: Supplementary file 3 — Table S2: Analysis of deviance comparing nested Cox proportional hazards models that incorporate OD, pLSC6 score, and Immunophenotype versus OD, pLSC6 score, Immunophenotype, and Transcriptomic Diversity (HDOD or LDOD). NA, Not Applicable. [file CAM4-14-e71325-s001.docx]

| Analysis of Deviance Table | | | | | |
| --- | --- | --- | --- | --- | --- |
| Model | Formula | Log-Likelihood | Chi-Square | Degrees of Freedom | p-value |
| Model 1 | Oncogenic Driver + pLSC6 + Immunophenotype | −869.46 | NA | NA | NA |
| Model 2 | Oncogenic Driver + pLSC6 + Immunophenotype + Transcriptomic Diversity | −869.46 | 0.00 | 0 | 1.00 |

**Supplementary Table 2.** Analysis of deviance comparing nested Cox proportional hazards models that incorporate OD, pLSC6 score, and Immunophenotype vs. OD, pLSC6 score, Immunophenotype, and Transcriptomic Diversity (HDOD or LDOD). NA, Not Applicable
