# Supplementary material for: Transcriptomic Diversity of Pediatric Acute Myeloid Leukemia Genetic Drivers Correlates With Clinical Outcome and Expression of Stemness‐Related Genes
Source: Cancer Med. 2025 Nov 3;14(21):e71325. doi: 10.1002/cam4.71325 (PMC12580620; doi:10.1002/cam4.71325)
Supplement: Supplementary file 4 — Table S3: Model comparison using Akaike Information Criterion (AIC). [file CAM4-14-e71325-s004.docx]

| Model Comparison using AIC | | |
| --- | --- | --- |
| Model | Degrees of Freedom | AIC |
| Transcriptomic Diversity + pLSC6 + Immunophenotype | 8 | 1,806.89 |
| Oncogenic Driver + pLSC6 + Immunophenotype | 28 | 1,794.93 |

AIC: Akaike information criterion

**Supplementary Table 3.** Model comparison using Akaike Information Criterion (AIC).
